# Internalization of Muscle-Specific Kinase Is Increased by Agrin and Independent of Kinase-Activity, Lrp4 and Dynamin

**DOI:** 10.3389/fnmol.2022.780659

**Published:** 2022-03-15

**Authors:** Anna Gemza, Cinzia Barresi, Jakob Proemer, Jasmin Hatami, Margarita Lazaridis, Ruth Herbst

**Affiliations:** Center for Pathophysiology, Infectiology and Immunology, Medical University of Vienna, Vienna, Austria

**Keywords:** neuromuscular junction, endocytosis, receptor tyrosine kinase, MuSK, signal transduction, skeletal muscle

## Abstract

Muscle-specific kinase (MuSK) is a receptor tyrosine kinase absolutely required for neuromuscular junction formation. MuSK is activated by binding of motor neuron-derived Agrin to low-density lipoprotein receptor related protein 4 (Lrp4), which forms a complex with MuSK. MuSK activation and downstream signaling are critical events during the development of the neuromuscular junction. Receptor tyrosine kinases are commonly internalized upon ligand binding and crosstalk between endocytosis and signaling has been implicated. To extend our knowledge about endocytosis of synaptic proteins and its role during postsynaptic differentiation at the neuromuscular junction, we studied the stability and internalization of Lrp4, MuSK and acetylcholine receptors (AChRs) in response to Agrin. We provide evidence that MuSK but not Lrp4 internalization is increased by Agrin stimulation. MuSK kinase-activity is not sufficient to induce MuSK internalization and the absence of Lrp4 has no effect on MuSK endocytosis. Moreover, MuSK internalization and signaling are unaffected by the inhibition of Dynamin suggesting that MuSK endocytosis uses a non-conventional pathway and is not required for MuSK-dependent downstream signaling.

## Introduction

The transmission of brain-derived electrical impulses to muscles depends on a specialized region referred to as the neuromuscular junction (NMJ). NMJs are interfaces formed between the presynaptic motor nerve terminals and the postjunctional muscle fibers that regulate skeletal muscle contraction (Sanes and Lichtman, 2001). Impaired communication between the presynaptic and postsynaptic compartments can lead to severe pathological disorders associated with the neuromuscular system, such as myasthenia gravis, congenital myasthenic syndromes and amyotrophic lateral sclerosis.

The development of the NMJ depends on the coordinated interplay between the receptor tyrosine kinase (RTK) MuSK, its co-receptor Lrp4, a member of the low-density lipoprotein (LDL) receptor family and the neurally secreted ligand Agrin (Burden et al., 2018; Li et al., 2018; Herbst, 2020). The binding of Agrin to Lrp4 leads to the formation of an Agrin-Lrp4 hetero-tetramer, thereby inducing the dimerization of MuSK and the subsequent autophosphorylation of specific tyrosine residues within its cytoplasmic region (Watty et al., 2000; Zong et al., 2012). While the ectodomain of MuSK is mainly responsible for the interaction with the accessory receptor Lrp4 and the ligand-dependent receptor dimerization, the intracellular region of MuSK plays a crucial role in the transduction of signals derived from the extracellular space. Phosphorylation of specific tyrosine residues in the intracellular portion of MuSK upon Agrin stimulation creates docking sites for cytoplasmic adaptor proteins (Herbst and Burden, 2000; Okada et al., 2006). An adaptor protein referred to as Dok-7 binds to the phosphorylated tyrosine residue Y553 and enhances MuSK activation, thereby acting as an intracellular MuSK activator (Bergamin et al., 2010). Once fully active, MuSK initiates downstream signaling that drives postsynaptic differentiation including the clustering of AChRs (Herbst, 2020).

As the existence of a close linkage between signaling and receptor trafficking was demonstrated for many RTKs, the investigation of molecular mechanisms regulating the endocytosis of MuSK is of particular importance for the understanding of MuSK signaling at the NMJ. Ligand binding usually drives the activation and subsequent endocytosis of RTKs into internal membrane compartments, thereby controlling the strength and specificity of the response (Sorkin and von Zastrow, 2009; Hupalowska and Miaczynska, 2012; Goh and Sorkin, 2013). However, the activation mechanism of MuSK differs considerably from those of conventional RTKs involving a multi-step process including Lrp4 binding for MuSK activation and Dok-7 recruitment for full kinase activity. Zhu and colleagues were able to provide evidence that MuSK endocytosis is stimulated upon Agrin treatment (Zhu et al., 2008). It was proposed that MuSK endocytosis is required for MuSK signaling since expression of dominant-negative Dynamin reduces Agrin-stimulated MuSK internalization and AChR clustering. Other reports demonstrated that MuSK turnover occurs independent of Agrin (Luiskandl et al., 2013). Given the sparse knowledge about endocytosis of synaptic proteins and its role during postsynaptic differentiation at the NMJ, we focused on the stability and endocytosis of Lrp4, MuSK and AChRs in response to Agrin. We present evidence that MuSK but not Lrp4 internalization is increased by Agrin stimulation. Strikingly, we find that neither the lack of Lrp4 nor constitutive kinase-activity affect MuSK internalization. Finally, inhibition of Dynamin-2 does not block Agrin-induced internalization and downstream signaling.

## Materials and Methods

### Reagents

The following antibodies were purchased from commercial sources: anti-phospho-tyrosine PY20 (BD Biosciences, Franklin Lakes, NJ, United States), PY99 (Santa Cruz Biotechnology), P-Tyr-100 (Cell Signaling Technology, Beverly, MA, United States), anti-AChRα (BD Biosciences, Franklin Lakes, NJ, United States), anti-AChRβ (Sigma-Aldrich, St. Louis, MO, United States), anti-MuSK AF562 (R&D Biosystems, Minneapolis, MN, United States), anti-Lrp4 (Abcam, Cambridge, United Kingdom), anti-Actin (BD Biosciences, Franklin Lakes, NJ, United States), anti-insulin receptor β (BD Biosciences, Franklin Lakes, NJ, United States). Biotin- and Alexa 594-conjugated α-bungarotoxin (BGT) were obtained from Invitrogen. Secondary HRP-conjugated antibodies were purchased from Jackson ImmunoResearch, Ely, United Kingdom. For immunoprecipitation antibodies against the C-terminal sequence of MuSK were used as described previously (Herbst and Burden, 2000; Bergamin et al., 2010). Streptavidin and Protein A agarose beads were obtained from Thermo Fisher Scientific, Waltham, MA, United States. Soluble neural A4B8 Agrin was prepared from HEK 293T as reported elsewhere (Herbst and Burden, 2000). The inhibitors Dyngo-4a was purchased from Cayman Chemicals, Ann Arbor, MI, United States and Dynasore as well as Primaquine were obtained from Sigma-Aldrich, St. Louis, MO, United States. Cycloheximide was purchased from Roche Diagnostics, Mannheim, Germany.

### Cell Culture

C2C12 myoblasts were originally obtained from the laboratory of Dr. Steven J. Burden (NYU School of Medicine, NYC, United States). Cells were grown and differentiated as according to previously described protocols (Nizhynska et al., 2007). Immortalized wild-type muscle cells were generated from limb muscles of E18 C57/Bl6;H-2Kb-tsA58 embryos as previously described (Herbst and Burden, 2000). MuSK^–/–^ muscle cells expressing MuSK wild-type, MuSK kinase-active (KA) or MuSK kinase-dead (KD) were generated and described previously (Mazhar and Herbst, 2012). Cells were cultured in Dulbecco’s Modified Eagle’s Medium (DMEM) with 4.5 mg/ml glucose, 10% (v/v) fetal bovine serum (Hyclone), 10% (v/v) horse serum (HS), 20 u/ml recombinant mouse interferon γ (PeproTech, London, United Kingdom), 1% (v/v) penicillin/streptomycin at 33°C and 5% CO_2_. To induce differentiation cells were shifted to DMEM with 10% (v/v) HS and 1% (v/v) penicillin/streptomycin at 37°C and 5% CO_2_. Differentiated cells were preincubated with 15 μg/ml cycloheximide, 200 μM Primaquine, 30 μM Dyngo-4a or 80 μM Dynasore for 30 min, and stimulated with 5 nM neural Agrin for the indicated time periods. Inhibitors were pre-diluted in DMEM prior to dropwise addition.

### Generation of C2/Lrp4-KO Muscle Cells

Two single guide RNAs (sgRNA) were designed to target exon 2 and exon 3 of the murine *lrp4* gene (Exon 2: 5′-GTACCTGTATCCCGCCCAG-3′; Exon 3: 3′-CGTCACACACCCAGGAGCGT-5′). The sgRNAs were co-delivered with Cas9-nuclease, using two different plasmids – either pSpCas9(BB)-2A-GFP (PX458) or pSpCas9(BB)-2A-Puro (PX459) purchased from Addgene, Cambridge, MA, United States (Ran et al., 2013). C2C12 myoblasts were transfected with different constructs using Turbofect (Thermo Fisher Scientific, Waltham, MA, United States). Myoblasts transfected with the PX458 plasmid were sorted by fluorescence activated cell sorting for GFP and myoblasts transfected with the PX459 construct were selected in growth medium containing puromycin for 48 h. Single cell clones were isolated and assayed for fusion and Lrp4 expression. Three clones were selected for further characterization (Supplementary Figure 1). Sequence analysis of the targeted genomic region identified deletions and insertions that cause frameshifts and premature termination of translation due to resulting stop codons. Two different clones both targeting exon 3 (3#2 and 3#9) were used for the analysis of MuSK surface expression.

### Biotinylation of Surface Proteins

Cells were labeled with 0.5 mg/ml Sulfo-NHS-LC-biotin (Pierce, Rockford, IL, United States) in PBS with 1mM CaCl_2_ and 1 mM MgCl_2_ at 4°C for 30 min. After washing two times with PBS/CaCl_2_/MgCl_2_, the unbound biotin was quenched three times with 50 mM glycine in PBS/CaCl_2_/MgCl_2_ for 5 min. Cells were then rinsed with PBS/CaCl_2_/MgCl_2_ and lysed in RIPA buffer [50 mM Tris-HCl pH 7.5, 150 mM NaCl, 1 mM EDTA, 1% (v/v) NP-40, 0.5% (v/v) DOC, 0.1% (v/v) SDS] supplemented with protease inhibitors [0.2 mM phenylmethylsulfonyl fluoride (PMSF), 1 μg/ml aprotinin, 1 μg/ml leupeptin, 1 μg/ml pepstatin] and phosphatase inhibitors (1 mM sodium orthovanadate, 50 mM sodium fluoride), 0.5% (v/v) SDS and 0.1 μl/ml nuclease (Pierce Universal Nuclease for Cell lysis, Thermo Fisher Scientific, Waltham, MA, United States) for 15 min at 4°C. Cells were scraped off the plate and centrifuged at full speed for 15 min at 4°C. Aliquots were collected to detect total protein concentrations and the remaining lysates were incubated with streptavidin agarose overnight at 4°C. After washing three times with RIPA buffer containing protease inhibitors, bound proteins were eluted from the beads with sample buffer and analyzed by immunoblotting.

### Immunoprecipitation, Acetylcholine Receptor Pulldown, and Immunoblotting

Differentiated myotubes were starved for 2 h in DMEM, pretreated with inhibitors and stimulated with neural Agrin. Cells were lysed in RIPA buffer supplemented with protease and phosphatase inhibitors. Samples were centrifuged 15 min at 4°C and the supernatant was used to isolate MuSK and AChRs. Proteins were subjected to SDS-PAGE followed by immunoblotting. Primary antibody incubations were performed over night at 4°C. After incubation with secondary antibodies, signals were detected *via* chemiluminescence (Millipore Sigma, St. Louis, MO, United States). Images were acquired using a Bio-Rad ChemiDoc (Bio-Rad Laboratories).

### Acetylcholine Receptor Clustering

To induce AChR clustering, myotubes were stimulated for 7 h with neural Agrin with and without Dyngo-4a or Dynasore. To label surface AChRs, cells were fixed with 2% PFA in PBS at room temperature, washed two times with PBS and incubated with Alexa 594-conjugated α-BGT in 2% FBS/PBS for 30 min. Cells were washed three times with PBS for 5 min and mounted with Mowiol 4-88. AChR clusters were imaged with a LEICA DM-IRB fluorescence microscope (40x oil immersion magnification objective) using Metamorph software (Molecular Devices, San Jose, CA, United States).

### Quantification and Statistics

Immunoblots were analyzed using Image Lab Software (Bio-Rad Laboratories, Hercules, CA, United States). To quantify MuSK and AChRβ phosphorylation, the ratio of phosphorylated protein to total protein was quantified. Surface expression of MuSK, Lrp4 and AChRα was normalized against insulin receptor β (IRβ), which served as control for biotinylation since it is unaffected by Agrin treatment. Total protein expression was normalized against Actin. Within each experiment time point 0 was defined as 1.0 and used to calculate the relative increase for the other time points. Number of experiments (n) refers to independent experiments performed using individual cell cultures. Detailed information about number of experiments and descriptive statistics are provided in Supplementary Table 1. AChR clusters were analyzed using an automated macro for the ImageJ software (Rodriguez Cruz et al., 2020). In each image the myotube area was selected and all objects with a minimum size of 8 μm^2^ were measured. AChR clustering experiments were replicated in at least four independent experiments (*n* = 4). At least 20 images per condition and experiment were analyzed. Data were assessed using the Kolmogorov-Smirnov test confirming normality of sample distribution. Data were analyzed using independent Samples *T*-test and parametric one-way ANOVA for two or more group comparisons and two-way ANOVA for two factor comparisons with Tukey’s multiple comparison tests. GraphPad Prism version 6.0 was used for analysis. The values of *p* < 0.05 (*), *p* < 0.01 (^**^), *p* < 0.001 (^***^) and *p* < 0.0001 (^****^) were considered statistically significant.

## Results

### Muscle-Specific Kinase and Acetylcholine Receptor Endocytosis but Not Low-Density Lipoprotein Receptor Related Protein 4 Endocytosis Is Increased by Agrin

**FIGURE 1:**
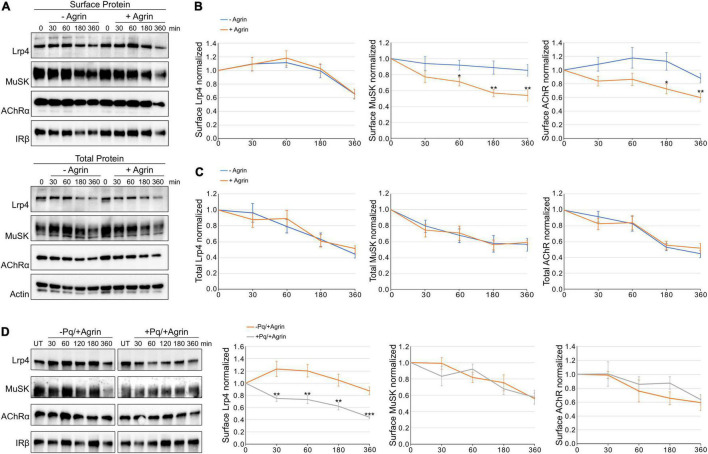
Agrin stimulation increases MuSK and AChR internalization but does not affect Lrp4 internalization. Muscle cells were pretreated with cycloheximide and incubated with or without 5 nM Agrin for the indicated time periods (in minutes). Surface proteins were isolated using biotinylation followed by streptavidin pulldown. **(A)** Samples were subjected to SDS-PAGE and analyzed by immunoblotting. Surface and total protein expression was determined using antibodies against Lrp4, MuSK, AChRα, IRβ and Actin. **(B)** Quantification of surface protein expression as function of time and Agrin treatment (− Agrin, blue; + Agrin, orange) is shown. Isolated surface proteins were normalized against IRβ. Timepoint 0 was set to 1. Data are presented as means ± SEM; *n* > 9. **(C)** Quantification of total protein expression as function of time and Agrin treatment (− Agrin, blue; + Agrin, orange). Total protein samples were normalized against Actin. Timepoint 0 was set to 1. Data are presented as means ± SEM; *n* > 5. **(D)** Muscle cells were pretreated with cycloheximide in the presence or absence of Primaquine (Pq) followed by stimulation with 5 nM Agrin for the indicated time periods (in minutes). Surface proteins were isolated using biotinylation followed by streptavidin pulldown. Samples were subjected to SDS-PAGE and analyzed by immunoblotting. Quantification of surface protein expression as function of time and Primaquine treatment (− Primaquine, orange; + Primaquine, gray) is shown. Isolated surface proteins were normalized against IRβ. Timepoint 0 was set to 1. Data are presented as means ± SEM; *n* > 3. *p < 0.05; **p < 0.01; ***p < 0.001.

Zhu et al. (2008) previously demonstrated a fast increase of MuSK internalization in response to Agrin but no effect on AChR internalization. In contrast, we have previously shown that MuSK turn-over is not affected by Agrin treatment (Luiskandl et al., 2013). To study the role of Agrin in protein endocytosis in more detail, we determined surface protein expression of AChR, Lrp4, and MuSK upon Agrin stimulation. For this, muscle cells were pretreated with cycloheximide and stimulated with Agrin for different time periods. We analyzed surface expression in response to Agrin using cell surface biotinylation followed by immunoblotting (Figure 1A). Quantitative analysis of Lrp4, MuSK and AChR expression revealed that surface expression of all proteins was very stable over the course of 6 h in the absence of Agrin. Lrp4 and AChR even showed a transient increase of surface expression between 30 and 60 min. It is known that AChRs are tightly associated with cytoskeletal proteins, which may stabilize surface AChRs (Dai et al., 2000; Dobbins et al., 2006), whether that is also the case for Lrp4 is unknown. In the presence of Agrin, MuSK and AChR endocytosis was increased for all time points compared to untreated samples and surface expression was decreased to around 60% after 6 h (compared to around 90% in untreated cells). In contrast, Lrp4 endocytosis and surface expression was not affected by Agrin treatment (Figure 1B). Next, we also studied protein stability in muscle cells pretreated with cycloheximide and stimulated with Agrin (Figure 1A). Quantification of protein expression showed that Lrp4, MuSK and AChR stability is unaffected by Agrin stimulation (Figure 1C). Total protein expression in the both, absence and presence of Agrin was decreased to 40–60% after 6 h. Additionally, we evaluated the contribution of recycling to the amount of surface protein by using Primaquine, which inhibits the recycling of endocytosed proteins to the plasma membrane (van Weert et al., 2000). We treated cells with cycloheximide in the presence or absence of Primaquine prior to stimulation with Agrin. Surface proteins were isolated and analyzed by immunoblotting. The quantification of surface proteins showed that recycling does not play a role in the case of MuSK indicating that MuSK is not recycled upon internalization (Figure 1D). In contrast, surface levels of Lrp4 are significantly higher in samples treated only with cycloheximide compared to cells treated with cycloheximide and Primaquine, implying that Lrp4 is recycled back to the cell surface over the time course of 6 h. AChR surface expression is not affected by Primaquine, which is consistent with a previous report demonstrating a lack of AChR recycling in cultured muscle cells (Brenner and Akaaboune, 2014). Taken together, we found that MuSK and AChR endocytosis was increased in response to Agrin whereas Lrp4 internalization was Agrin-independent. Moreover, MuSK and AChRs are not recycled upon internalization whereas Lrp4 is recycled back to the cell surface.

### Muscle-Specific Kinase Activity Does Not Increase Muscle-Specific Kinase Endocytosis

**FIGURE 2:**
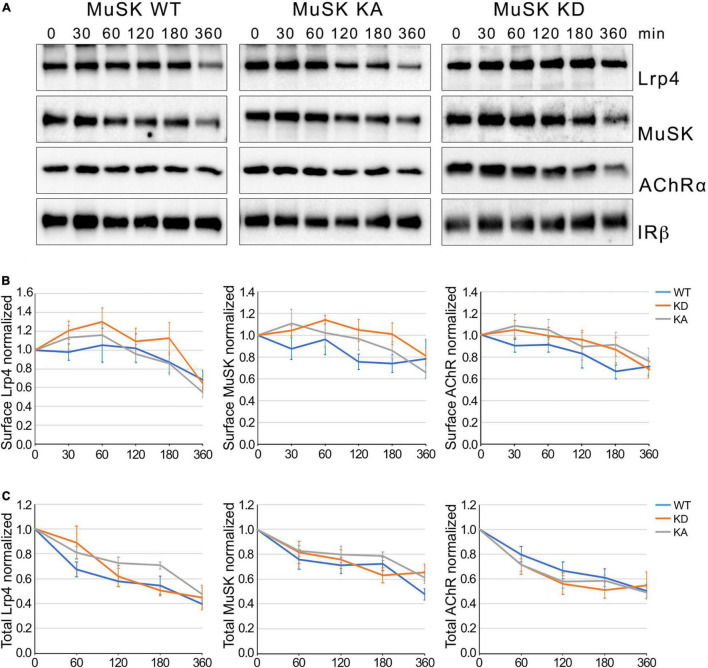
MuSK kinase activity fails to induce MuSK internalization. **(A)** Muscle cells expressing MuSK wildtype (WT), MuSK kinase-active (KA) or MuSK kinase-dead (KD) were treated with cycloheximide for the indicated time periods (in minutes). Surface proteins were isolated using biotinylation followed by streptavidin pulldown. Protein expression was determined by immunoblotting using antibodies against Lrp4, MuSK, AChRα and IRβ. **(B)** Quantification of surface protein expression in muscle cells expressing MuSK wildtype (blue), kinase-active (gray) and kinase-dead (orange) is shown over a time course of 6 h. Isolated surface proteins were normalized against IRβ. Timepoint 0 was set to 1. Data are presented as means ± SEM, *n* > 5. **(C)** Quantification of total protein expression as function of time. Total protein samples of MuSK wildtype (blue), kinase-active (gray) and kinase-dead (orange) were normalized against Actin. Timepoint 0 was set to 1. Data are presented as means ± SEM; *n* > 5.

Ligand-induced activation of RTKs has been reported as an important regulatory event for receptor internalization (Goh and Sorkin, 2013; Miaczynska, 2013). Phosphorylation of the intracellular residues is able to act as signal for internalization by recruiting factors of the endocytic machinery. To determine whether MuSK kinase activation drives MuSK endocytosis we use muscle cells expressing either MuSK wildtype (WT), MuSK kinase-active (KA) or MuSK kinase-dead (KD). In previous studies, we have shown that MuSK-KA is able to induce MuSK activation and AChR clustering independent of Agrin whereas MuSK-KD is unresponsive to Agrin and fails to induce MuSK activity and subsequent signaling (Mazhar and Herbst, 2012). Myotubes were treated with cycloheximide for different time periods and surface protein expression was analyzed by surface biotinylation followed by immunoblotting (Figure 2A). Quantitative analysis of Lrp4, MuSK and AChR expression showed that surface expression of all proteins decreased slowly over the time course of 6 h, similar to the surface expression observed in wild-type muscle cells (Figure 1). Interestingly, we found no difference in surface expression between MuSK-KA and MuSK-WT or MuSK-KD (Figure 2B). Next, we also studied protein stability in myotubes treated with cycloheximide by analyzing total protein expression over the time course of 6 h (Supplementary Figure 2 and Figure 2C). As shown in Figure 2C, Lrp4, MuSK and AChR expression is not affected by MuSK kinase-activity. Protein expression in all cell lines was decreased to 40–60% after 6 h. We conclude from these experiments that MuSK kinase activity does not induce MuSK endocytosis or affect endocytosis of Lrp4 and AChR. Further, MuSK activity does not modulate protein stability.

### Muscle-Specific Kinase Endocytosis Is Independent of Low-Density Lipoprotein Receptor Related Protein 4

**FIGURE 3:**
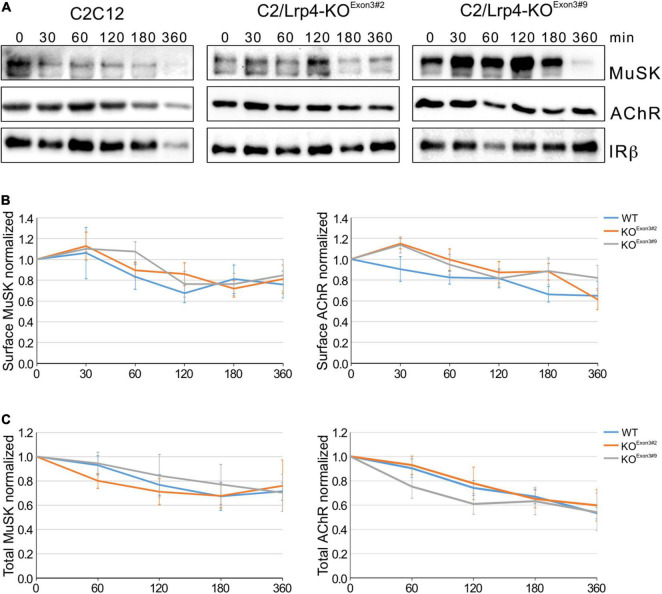
MuSK endocytosis occurs independent of Lrp4. **(A)** Wild-type C2 cells and C2/Lrp4-KO cells were treated with cycloheximide for the indicated time points (in minutes). Biotinylation was used to isolate surface proteins followed by analysis by immunoblotting using antibodies against MuSK, AChRα and IRβ. **(B)** Quantification of MuSK and AChR surface protein expression in C2 wild-type cells (blue) and C2/Lrp4-KO cells (Exon3#2: orange; Exon3#9: gray) is shown over a time course of 6 h. Isolated surface proteins were normalized against IRβ. Timepoint 0 was set to 1. Data are presented as means ± SEM, *n* > 4. **(C)** Quantification of total protein expression as function of time in C2 wild-type cells (blue) and C2/Lrp4-KO cells (Exon3#2: orange; Exon3#9: gray). Total protein samples were normalized against Actin. Timepoint 0 was set to 1. Data are presented as means ± SEM; *n* > 4.

Low-density lipoprotein receptor related protein 4 belongs to the family of lipoprotein receptor-related family. Its founding member Lrp1 is a well characterized membrane protein involved in receptor-mediated endocytosis, thereby targeting binding partners either to lysosomes or recycling endosomes. To study the role of Lrp4 in MuSK endocytosis we generated C2 muscle cells lacking Lrp4 using CRISPR/Cas9. These cells fail to form AChR clusters in response to Agrin and MuSK is not phosphorylated in the presence of Agrin (Supplementary Figure 1). C2/Lrp4-KO and C2 wild-type myotubes were treated with cycloheximide for different time points, surface proteins were labeled with biotin and isolated using streptavidin-agarose. Surface expression of proteins was analyzed by immunoblotting (Figure 3A). Quantification of MuSK and AChR surface expression revealed a slow decrease of expression over the time course of 6 h and no difference in surface expression between C2 wild-type cells and Lrp4 deficient C2 cells was observed (Figure 3B). The degree of decrease was similar to the surface expression observed in wild-type muscle cells in the absence of Agrin (Figure 1). We also studied protein stability in muscle cells treated with cycloheximide by analyzing total protein expression over the time course of 6 h (Supplementary Figure 3). Figure 3C shows that MuSK and AChR expression were not altered in the absence of Lrp4. These experiments demonstrate that while Lrp4 acts a binding partner of MuSK even in the absence of Agrin, Lrp4 does not influence MuSK internalization and stability.

### Low-Density Lipoprotein Receptor Related Protein 4, Muscle-Specific Kinase and Acetylcholine Receptor Internalization Occurs Dynamin-Independent

**FIGURE 4:**
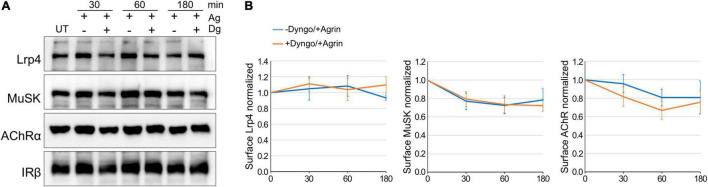
Lrp4, MuSK and AChR internalization takes place independent of Dynamin. Myotubes were pretreated with cycloheximide with or without Dyngo-4a and subsequently stimulated with 5 nM Agrin for the indicated time periods (in minutes). Surface proteins were isolated using biotinylation followed by streptavidin pulldown. **(A)** Samples were subjected to SDS-PAGE and analyzed by immunoblotting. Surface expression was determined using antibodies against Lrp4, MuSK, AChRα and IRβ. **(B)** Quantification of surface protein expression as function of time and Dyngo-4a treatment (− Dyngo-4a, blue; + Dyngo-4a, orange) is shown. Isolated surface proteins were normalized against IRβ. Timepoint 0 was set to 1. Data are presented as means ± SEM; *n* > 4. Ag, Agrin; Dg, Dyngo-4a; UT, untreated.

Zhu et al. (2008) have shown that expression of a dominant-negative mutant of Dynamin-2 in C2 muscle cells blocks Agrin-induced MuSK internalization and subsequent AChR clustering. Similar, we have demonstrated in heterologous cells that Agrin-independent MuSK internalization is decreased by the Dynamin-inhibitor Dynasore (Luiskandl et al., 2013). To follow up on these studies, we determined the rate of AChR, Lrp4 and MuSK internalization in myotubes, when Dynamin-dependent endocytosis is blocked. We pretreated muscle cells with cycloheximide and Dyngo-4a, an inhibitor of Dynamin with more potency and higher specificity than Dynasore (Supplementary Figure 4; McCluskey et al., 2013) followed by stimulation with Agrin for different time periods. Surface proteins were biotinylated, isolated using streptavidin-agarose and analyzed by immunoblotting (Figure 4A). Protein expression was quantified (Figure 4B). As described above, Agrin stimulated MuSK and AChR internalization but did not affect Lrp4 internalization. Surprisingly, pretreatment of cells with Dyngo-4a had no effect on Lrp4, MuSK or AChR internalization over the time period of 3 h. Similarly, Dynamin-inhibition did not affect Lrp4, MuSK and AChR internalization in the absence of Agrin (Supplementary Figure 5).

**FIGURE 5:**
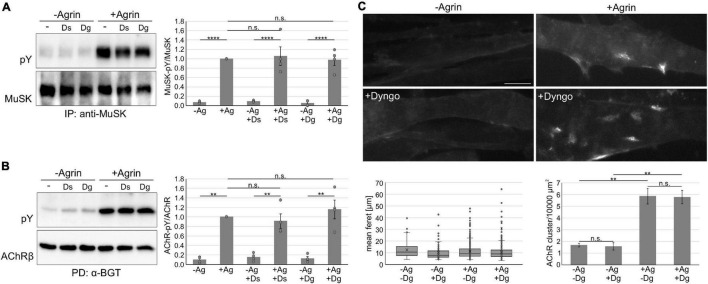
Inhibition of dynamin does not affect MuSK activation and signaling. **(A)** Myotubes were pretreated with Dyngo-4a, Dynasore or vehicle (DMSO) and subsequently incubated without or with 5 nM Agrin for 30 min. MuSK was isolated from cell lysates and phosphorylation and expression were determined using antibodies against phospho-tyrosine and MuSK, respectively. MuSK phosphorylation normalized to MuSK expression was quantified and samples treated with Agrin in the absence of inhibitor were set to 1. Data are presented as means ± SEM; *n* = 4. **(B)** Myotubes were pretreated with Dyngo-4a, Dynasore or vehicle (DMSO) and subsequently incubated without or with 5 nM Agrin for 60 min. AChRs were isolated from cell lysates. Phosphorylation and expression were determined using antibodies against phospho-tyrosine and AChRβ, respectively. AChRβ phosphorylation normalized to AChRβ expression was quantified and samples treated with Agrin in the absence of inhibitor were set to 1. Data are presented as means ± SEM; *n* = 4. **(C)** Myotubes were pretreated with Dyngo-4a or vehicle (DMSO) and subsequently incubated without or with 0.5 nM Agrin for 7 h. AChRs were stained with Alexa 594-conjugated α – BGT and analyzed by microscopy. Representative images are shown. The feret and number of clusters were analyzed using ImageJ. Scale bar = 20 μm. *n* = 4. Ag, Agrin; Dg, Dyngo-4a; Ds, Dynasore; IP, immunoprecipitation; PD, pulldown; PY, phospho-tyrosine. **p < 0.01; ****p < 0.0001; n.s., non significant.

We next analyzed MuSK and AChR phosphorylation in myotubes treated with Dyngo-4a followed by a stimulation with Agrin. MuSK and AChRs were isolated from cell lysates and their phosphorylation studied by immunoblotting (Figure 5A). Quantitative analysis revealed that MuSK activation and downstream signaling occurred independent of Dynamin (Figure 5B). MuSK activation results in the clustering of AChRs, which involves a complex signaling cascade that is not yet fully resolved. To study the role of Dynamin in AChR clustering, we pretreated myotubes with Dyngo-4a and subsequently stimulated with Agrin. Cells were stained with fluorescently-labeled α-BGT and analyzed by microscopy (Figure 5C). Consistent with our data showing Dynamin-independent internalization and signaling, we observed similar numbers of AChR clusters as well as similar cluster sizes in myotubes treated and untreated with Dyngo-4a.

Based on these experiments we propose that Dynamin-dependent processes do not play a role during Agrin-induced internalization and signaling.

## Discussion

Receptor endocytosis represents an important mechanism to regulate receptor signaling. Little is known about MuSK endocytosis and its role in MuSK signaling. Our study provides a comprehensive analysis of surface expression and stability of Lrp4 and MuSK upon Agrin stimulation. Our results imply that Agrin-induced MuSK signaling occurs at the membrane and does not trigger a fast MuSK endocytosis but rather a slow decrease of MuSK surface protein. MuSK kinase-activity alone is not sufficient to drive MuSK internalization. Furthermore, we exclude a role of Dynamin in MuSK endocytosis and signaling. We observe rapid recycling of Lrp4, but the absence of Lrp4 does not affect MuSK internalization.

Ligand-induced activation of most RTKs increases the rate of RTK internalization and ultimately leads to their downregulation. Thus, ligand-induced endocytosis attenuates the strength and duration of cell surface signaling by reducing the concentration of surface RTKs (Sorkin and von Zastrow, 2009; Miaczynska, 2013). The mechanism of MuSK activation differs considerably from those of conventional RTKs since MuSK is not activated by direct ligand-binding but instead Lrp4 is required for MuSK activation and dimerization, and full kinase activity of MuSK is only achieved upon binding of the intracellular adaptor protein Dok-7 (Herbst, 2020). MuSK activation therefore constitutes a multi-step process that involves MuSK interaction with multiple proteins. In the present study, we find an increase of MuSK internalization upon stimulation with Agrin. Internalization was slow and reached 40% after 6 h of Agrin treatment versus 20% without Agrin. A previous study by Zhu et al. reported that endocytosis of MuSK was increased by 36% after 30 min of Agrin treatment compared to unstimulated controls (later time points were not tested) (Zhu et al., 2008). Zhu and colleagues measured MuSK endocytosis by studying the level of internalized proteins in response to stimulation. Our experimental strategy determined the amount of protein on the cell surface at a given time point. Excluding recycling as contributing factor to MuSK surface expression, we conclude that MuSK endocytosis is increased after ligand activation. Although both studies report different internalization rates, which might be attributed to the different experimental designs and stimulation periods, our results are in accordance with findings reported by Zhu et al. (2008).

It has been proposed that an interplay between surface retention and endocytosis-promoting events determines the magnitude of RTK internalization caused by activation (Goh and Sorkin, 2013). Fast internalization is often seen with RTKs that can freely diffuse in the plasma membrane. In contrast, RTKs associated with cytoskeletal elements are immobile and fail to rapidly internalize (Mineo et al., 1999; Foti et al., 2004; Hommelgaard et al., 2004). Agrin-stimulation induces not only MuSK activation but also the clustering of MuSK to high-density patches on the muscle membrane. Cytoskeletal rearrangements and anchoring have been implicated in the Agrin-induced clustering process (Durnberger et al., 2014). The slow internalization of MuSK therefore might be a consequence of MuSK being retained in the plasma membrane *via* Agrin-induced clustering. Taking in consideration that cell type, expression and linkage to the plasma membrane influence endocytosis, this might also explain the contrasting fast constitutive internalization of MuSK observed in COS-7 cells (Luiskandl et al., 2013).

Ligand-induced activation results in autophosphorylation of tyrosines in the cytoplasmic region of RTKs. Phosphorylated residues function as recruitment sites for proteins that either link directly to the endocytic machinery or mediate ubiquitination, which then serves as sorting signal (Platta and Stenmark, 2011; Goh and Sorkin, 2013). Consistent with these findings it has been observed that kinase activity is required for ligand-dependent internalization (McClain, 1992). Similarly, it was shown that MuSK is ubiquitinated in response to Agrin and that surface expression of wild-type and kinase-active MuSK is decreased, when co-expressed with the E3 ubiquitin ligase PDZRN3 (Lu et al., 2007). We have used a constitutive-active MuSK mutant, which has previously been shown to induce downstream signaling independent of Agrin to study the role of kinase activity during MuSK internalization (Jones et al., 1999; Mazhar and Herbst, 2012). To our surprise, we found no significant difference in extent and kinetics of internalization between active MuSK, wild-type or kinase-dead MuSK. To our knowledge Agrin-dependent interactions of MuSK with known effectors of endocytosis have not been observed so far. The above mentioned PDZRN3 also binds to kinase-dead MuSK (Lu et al., 2007). Likewise, the binding of the vesicle-fusing ATPase/NSF, a protein proposed to regulate MuSK endocytosis, is independent of MuSK phosphorylation (Zhu et al., 2008). Therefore, it remains unclear how Agrin induces MuSK internalization but kinase-activity alone appears to be insufficient to increase internalization.

Clathrin-mediated endocytosis (CME) is the major pathway of internalization for ligand-bound RTKs. Experimental evidence demonstrates that essentially all members of the RTK family undergo CME but at the same time there is also growing support showing Clathrin-independent endocytosis (CIE; Sigismund et al., 2005; May et al., 2007; Haugsten et al., 2011; Reif et al., 2016). CME has been implicated in MuSK endocytosis since dominant-negative Dynamin interfered with Agrin-induced MuSK internalization (Zhu et al., 2008). Similarly, inhibition of Dynamin decreased constitutive internalization in COS-7 cells (Luiskandl et al., 2013). Surprisingly, in our hands Agrin-stimulated MuSK internalization was not affected using Dyngo-4a, a potent and specific Dynamin inhibitor. Correspondingly, MuSK signaling was also unaffected. We exclude a technical problem since the activity of Dyngo-4a was controlled by studying transferrin receptor internalization. Previously, we have reported CIE *via* Arf6 as potential mechanism of MuSK endocytosis in COS-7 cells (Luiskandl et al., 2013). This pathway is not well understood and experimental tools to inhibit this pathway specifically are limited (Donaldson et al., 2009; Donaldson and Jackson, 2011). It also has to be pointed out that RTKs can switch between CME and CIE and can use both pathways depending on ligand-concentration and cell type (Goh and Sorkin, 2013). Further studies are therefore required to determine the exact route of MuSK internalization.

Interestingly, Lrp4 and MuSK surface expression appear to be regulated differently since Agrin stimulation does not affect Lrp4 internalization. Members of the LDL receptor family, in particular LDL receptor, Lrp1, Lrp2 and Lrp6, are implicated as endocytic receptors, which bind and deliver molecules to intracellular compartments such as lysosomes (van Kerkhof et al., 2005; May et al., 2007; Sakane et al., 2010). Consistent with these reports we demonstrated that Lrp4, but not MuSK and AChR, is rapidly recycled upon internalization. A high rate of recycling might also explain the observed stable, transiently even slightly increased, expression of surface Lrp4. All members of the LDL receptor family carry an intracellular NPXY motif that has the potential to mediate the coupling of these receptors to the endocytic machinery as well as downstream signaling cascades. The role of the NPXY motif in Lrp4 is still unclear (Kim et al., 2008; Zhang et al., 2008). Our data suggest that Lrp4 internalization is not triggered by Agrin binding and that MuSK and Lrp4 do not internalize together. It has been reported that Lrp4 undergoes intramembraneous processing and shedding of the extracellular domain (Dietrich et al., 2010; Wu et al., 2012). Moreover, it was shown that the Lrp4 extracellular domain is sufficient to rescue AChR clustering in muscle cells and NMJ formation *in vivo* (Gomez and Burden, 2011; Wu et al., 2012). It therefore appears possible that Lrp4 is also removed by proteolytic cleavage. We did not observe a change in size or the appearance of cleavage products during the course of our experiments suggesting that proteolysis requires an additional signal besides Agrin. Therefore, how surface Lrp4 is regulated and whether Lrp4 also acts as endocytic receptor remains unresolved.

Our study was conducted with cultured muscle cells, which are a well-documented and still extensively used model for the examination and understanding of processes that occur at the postsynaptic side of the NMJ. Notably, the use of muscle cells does not fully reconstitute the complex structure of the NMJ *in vivo*, which involves a plethora of intertwined interactions between many cellular compartments, foremost the innervation of muscle fibers by motor neurons. Numerous reports have demonstrated that protein metabolism differs between intact muscle tissue and cultured muscle cells. In particular, nerve input appears to play a major regulatory role during protein trafficking (Rudolf and Straka, 2019). Of all proteins present at the NMJ, AChRs are the best studied with regard to endocytosis, recycling and degradation and it has been reported that these processes are regulated by the nerve (Rudolf and Straka, 2019; Martinez-Pena and Akaaboune, 2021). The question whether innervation also affects the trafficking of NMJ-associated proteins such as MuSK and Lrp4 remains so far elusive.

## Data Availability Statement

The raw data supporting the conclusions of this article will be made available by the authors, without undue reservation.

## Author Contributions

AG, CB, JP, and RH carried out the majority of experiments and data analyses. JH and ML contributed to the biochemical analysis of Lrp4 knock-out cells. RH formulated and oversaw the research project. AG and RH wrote the manuscript. All authors reviewed the manuscript.

## Conflict of Interest

The authors declare that the research was conducted in the absence of any commercial or financial relationships that could be construed as a potential conflict of interest.

## Publisher’s Note

All claims expressed in this article are solely those of the authors and do not necessarily represent those of their affiliated organizations, or those of the publisher, the editors and the reviewers. Any product that may be evaluated in this article, or claim that may be made by its manufacturer, is not guaranteed or endorsed by the publisher.
