# Supplementary material for: Internalization of Muscle-Specific Kinase Is Increased by Agrin and Independent of Kinase-Activity, Lrp4 and Dynamin
Source: Front Mol Neurosci. 2022 Mar 15;15:780659. doi: 10.3389/fnmol.2022.780659 (PMC8965242; doi:10.3389/fnmol.2022.780659)
Supplement: Supplementary file 1 [file Data_Sheet_1.PDF]

Supplementary Material

1 Supplementary Data

1.1 Transferrin receptor internalization

To evaluate the efficacy of the endocytic inhibitors, the internalization of Transferrin receptor was examined using tetramethylrhodamine isothiocyanate-labeled transferrin (TRITC-transferrin, Thermo Fisher Scientific, Waltham, MA). Briefly, NIH3T3 cells were cultured on gelatin-coated coverslips overnight. Cells were starved in serum-free DMEM for 2h and subsequently treated with 30 µM Dyngo-4a or 80 µM Dynasore for 30 min. Following the treatment, cells were incubated with 5 µg/ml TRITC-transferrin in the presence of inhibitors for 5 min at 37°C. After washing with ice-cold PBS once, TRITC-transferrin was stripped from the cell surface by washing twice with 0.5 M glycine (pH 2.2) for 1 min. Cells were rinsed with PBS and fixed in 4% PFA for 10 min at RT and mounted with Mowiol 4-88. The surface-stripped cells were visualized using a confocal microscope UltraVIEW ERS Rapid Confocal Imager (Perkin-Elmer) connected to a Zeiss Axiovert 200 microscope fitted with a 63x/1.4 oil objective lens (Plan-Apochromat, Zeiss).

2 Supplementary Figures and Tables

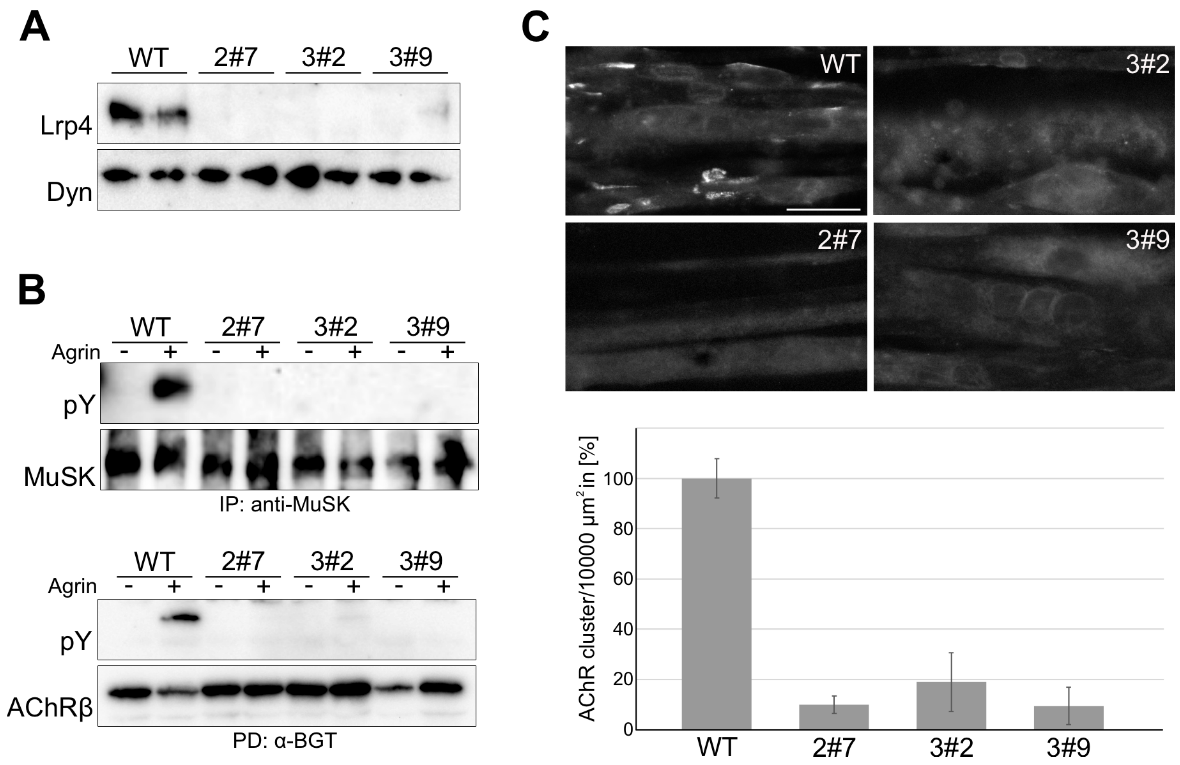

Supplementary Figure 1. C2C12 myotubes lacking Lrp4 fail to respond to Agrin stimulation.

Three selected clones of Crispr/Cas9 treated C2C12 were analyzed. 2#7: gRNA targeting exon 2 in PX459; 3#2: gRNA targeting exon 3 in PX459; 3#9: gRNA targeting exon 3 in PX458. (A) Myotubes were lysed and total protein samples were analyzed by immunoblotting using antibodies against Lrp4 and Dynamin-2. (B) Myotubes were stimulated with neural Agrin. After cell lysis, MuSK and AChRs were isolated from protein lysates. Samples were subjected to SDS-PAGE followed by immunoblotting. MuSK and AChRβ phosphorylation were analyzed by immunoblotting using antibodies against phospho-tyrosine. Blots were re-probed with anti-MuSK and anti-AChRβ antibodies, respectively. (C) Myotubes were stimulated with neural Agrin and AChRs were stained using Alexa594-conjugated α-BGT. Representative images are shown. Scale bar = 20 µm. IP, immunoprecipitation; PD, pulldown; pY, phospho-tyrosine; WT, wild-type C2C12.

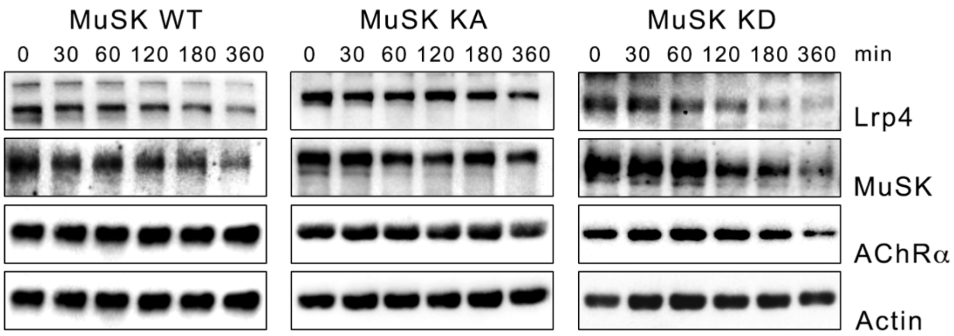

Supplementary Figure 2. MuSK kinase activity does not affect MuSK protein stability.

Muscle cells expressing MuSK wildtype (WT), MuSK kinase-active (KA) or MuSK kinase-dead (KD) were treated with cycloheximide for the indicated time periods (in minutes). Total protein expression was determined by immunoblotting using antibodies against Lrp4, MuSK, AChRα and Actin.

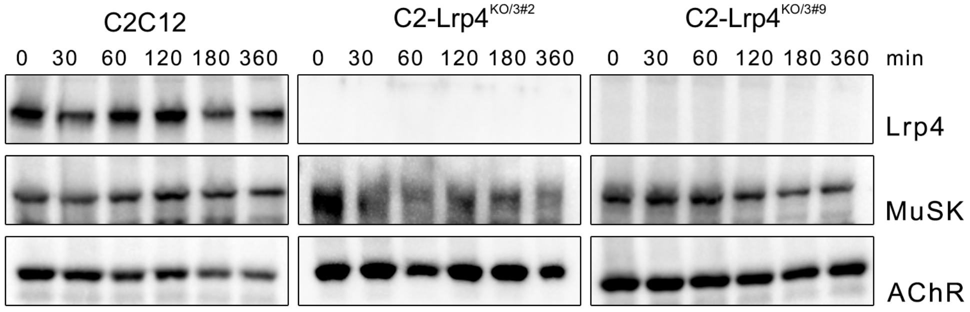

Supplementary Figure 3. The absence of Lrp4 does not affect MuSK protein stability.

C2C12 wildtype (WT) and C2-Lrp4-KO myotubes were treated with cycloheximide for the indicated time periods (in minutes). Protein expression was determined by immunoblotting using antibodies against Lrp4, MuSK and AChRα.

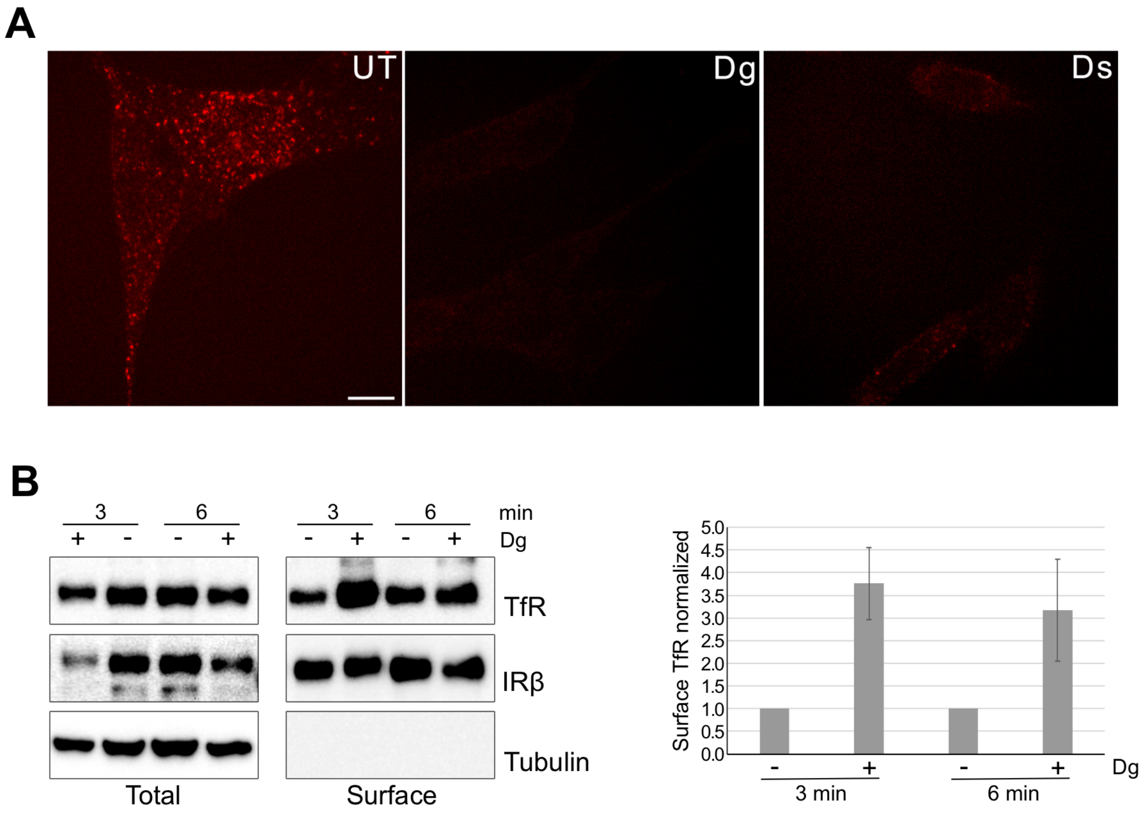

Supplementary Figure 4. Internalization of Transferrin receptor is blocked by Dyngo-4a and Dynasore.

(A) NIH 3T3 cells were treated with Dyngo-4a (Dg) or Dynasore (Ds) or left untreated (UT). TRITC-conjugated Transferrin was added to the cells for 5 minutes followed by removal of surface labeling using acidic washes. Representative images are shown. Scale bar = 15 µm. (B) NIH 3T3 cells were pretreated with Dyngo-4a or vehicle followed by stimulation with holo-Transferrin (5 µg/ml) for the indicated time. Surface proteins were biotinylated, isolated proteins subjected to SDS-PAGE and analyzed by immunoblotting using antibodies against TfR, IRβ and Tubulin. Surface protein expression was quantified and normalized against IRβ. Data are presented as means +/- SD.

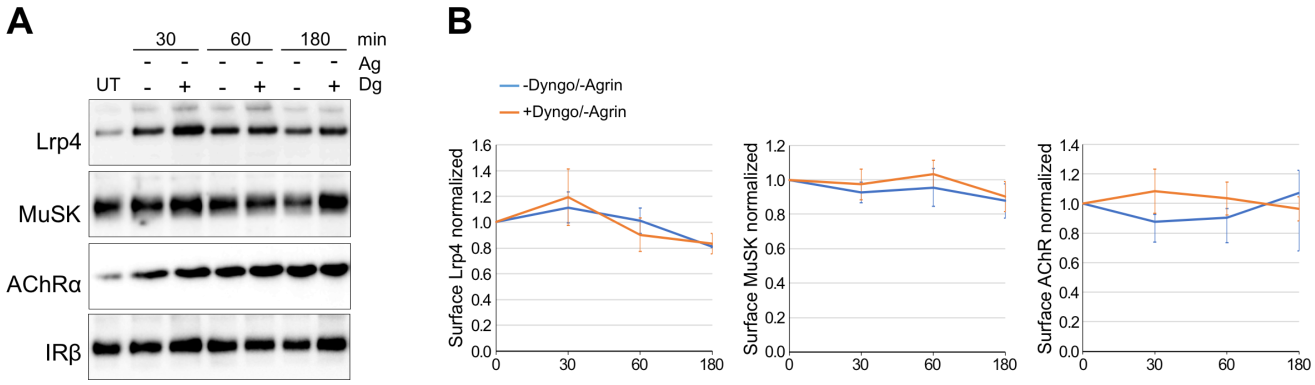

Supplementary Figure 5: Agrin-independent Lrp4, MuSK and AChR internalization in the presence of Dyngo-4a.

Myotubes were pretreated with cycloheximide with or without Dyngo-4a for the indicated time periods (in minutes). Surface proteins were isolated using biotinylation followed by streptavidin pulldown. (A) Samples were subjected to SDS-PAGE and analyzed by immunoblotting. Surface expression was determined using antibodies against Lrp4, MuSK, AChRα and IRβ. (B) Quantification of surface protein expression as function of time and Dyngo-4a treatment (- Dyngo-4a, blue; + Dyngo-4a, orange) is shown. Isolated surface proteins were normalized against IRβ. Timepoint 0 was set to 1. Data are presented as means +/- SEM; n ≧ 7. Ag, Agrin; Dg, Dyngo-4a; UT, untreated.
